# Public-Private Partnerships in Mexico: Implications of Engaging With the Food and Beverage Industry for Public Health Nutrition

**DOI:** 10.34172/ijhpm.2024.8008

**Published:** 2024-02-27

**Authors:** Angela Carriedo, Enaí Ojeda, Eric Crosbie, Mélissa Mialon

**Affiliations:** ^1^World Public Health Nutrition Association, London, UK.; ^2^Instituto Nacional de Salud Pública, Morelos, Mexico.; ^3^School of Public Health, University of Nevada Reno, Reno, NV, USA.; ^4^Ozmen Institute for Global Studies, University of Nevada Reno, Reno, NV, USA.; ^5^Trinity College Dublin, Dublin, Ireland.

**Keywords:** Public-Private Partnerships, Governance, Nutrition Programs

## Abstract

**Background:** In the last few years, Mexico adopted public health policies to tackle non-communicable diseases (NCDs), such as front of package nutrition labelling, food marketing restrictions to children, and a soda tax. In parallel, transnational food and beverage industries (F&BIs), their allies, and the government have agreed on public-private partnerships (PPPs) to implement policies or deliver programs. However, research has questioned the benefits of PPPs and exposed its limitations as a suitable mechanism to improve public health. This study analyses how four PPPs between the Mexican government, the F&BI, and allies are working to achieve their goals. We critically assessed the objectives, scope, reported impacts, governance principles and perceived risks and benefits for the public health agenda of these PPPs.

**Methods:** This qualitative study is based on 26 interviews with key actors, and 170 publicly available documents, including 22 obtained through freedom of information (FOI) requests related to four purposively selected PPPs aiming to improve health.

**Results:** We found that the four PPPs studied had minimal public information available on their implementation and impact. The private partners tend to dictate the design, information management, and implementation of the programs, while promoting their brands. Few independent evaluations of the PPPs exist, and none reported on their effectiveness or public health benefits. Good governance principles, such as accountability, transparency, fairness, participation, integrity, and credibility, were barely followed in each of the cases studied. Public officials did not automatically question the conflict of interest (CoI) of such arrangements. When there were COI, the potential risks these posed did not always outweigh the financial benefits of working with the F&BI and its allies.

**Conclusion:** The four PPPs studied produced minimal gains for public health while boosting credibility for the participating transnational F&BIs. It shows the lack of awareness of how these PPPs might be hindering public health gains.

## Background

**Key Messages**

**Implications for policy makers**

- Public-private partnerships (PPPs) between governments and the food and beverage industry (F&BI) and its allies should be questioned in terms of the potential negative implications they could have, notably because of the existence of a conflict of interest (CoI), a lack of transparency and good governance principles.
- Transparency, accountability, and ethical issues identified in the four PPPs under study are examples of issues to be considered at national and subnational levels to avoid the negative effects of partnering with transnational F&BI.
- Governments should reassess the mechanisms and tools in place to avoid CoIs and industry interference in public health program implementation when engaging in PPPs.

**Implications for the public**

Our results show a lack of synergy between the goals, aims, and actions described by the public and private partners involved in delivering the intended public health benefits in four public-private partnerships (PPPs). These PPPs show minimal gains for public health policy while boosting the credibility of the transnational food and beverage industry (F&BI) partners. PPPs are a corporate political activity aiming to minimise negative effects on transnational F&BI’s economic gains while being ‘part of the solution’ to the obesity crisis. These types of PPPs could negatively affect populations and governments. Advocates, policy-makers, and health professionals should be aware of these risks when considering partnerships with the F&BI.

The United Nations (UN) and the World Health Organization (WHO) have recommended Member States build strong coalitions as a key ingredient for tackling non-communicable diseases (NCDs) through policy change.^1-4^ This mandate is also part of other international obligations and recommendations such as the Sustainable Development Goals (SDGs), in particular SDG#17 on partnerships, and the UN Decade of Action on Nutrition.^5^ Given the lack of resources and state capacity in several low- and middle-income countries, public-private partnerships (PPPs) and multistakeholder arrangements (MSAs) have recently been seen as key collaborations towards building strong coalitions, as means of implementing programs and achieving public health goals. PPPs and MSAs constitute a hybrid type of governance in which non-state actors (eg, corporations) co-govern along with state actors to provide collective goods. These partnerships thereby adopt governance functions that have formerly been the sole authority of sovereign states.^6^ This transition from primarily public to a primarily public-private form of governing raises important new theoretical and political concerns of legitimacy^7^ and challenges existing conceptualisations of regulation.^8,9^ The increasing prominence of such arrangements^10-13^ raises questions about the identification and management of potential tensions between private partners and public health. PPPs and MSAs with unhealthy commodity industries, including the food and beverage industry (F&BI), have been shown to protect the industry’s economic interests more than promoting public health.^14^ PPPs and MSAs can neutralise the actions that public interested groups and governments might develop to improve public health policies.^15-17^ For instance, the involvement of commercial actors in multistakeholder consultations is likely to delay efforts to develop coherent political commitments to addressing the structural drivers of NCDs and other nutrition and food-related policy efforts.^11^

 Evaluations of PPPs in the field of public health nutrition have shown how the power of private partners exceeds those of others involved and facilitates these private entities to pursue business interests within the public health policy agenda. The F&BI acts as a legitimate participant in committees or advisory boards of health government agencies and has been seen as a legitimate partner for interventions aimed to improve community health,^18^ access to food or water, or promote behavioural change.^19^ However, there is also an increased recognition in the literature that the health agenda is so complex that no single sector or organisation can tackle it alone.^20,21^ While some authors have identified the limitations of hybrid governance, others argue that the complex agenda of NCDs requires a range of responses beyond the capacity of either the public or private sectors working independently, and therefore relationships need to be built between them.^22^

 PPPs are one of the many mechanisms of engagement between corporations, including transnational F&BI and different government agencies. PPPs have been classified as an instrumental action in relation to other tactics to obstruct or delay public health aims.^23,24^ Nevertheless, the transnational F&BI influence on the delivery and results of the programs delivered through PPPs is yet to be fully explored.^25^

###  Public Health Interventions and Public-Private Partnerships in Mexico

 In Mexico, the prevalence of obesity in adults is 75% and more than 35% schoolchildren are overweight, as rates of diabetes continue to escalate.^4^ Obesity was declared a national public health emergency in 2016, and again in 2018.^26,27^ More than 250 000 people die each year from causes related to NCDs, including heart disease and diabetes.^28^ These public health issues and changes in the countries’ obesity policy agenda have led to the proliferation of public policy actions and the implementation of programs to reduce NCDs with population interventions. Some of these interventions are outlined in the 2013’s National Strategy to Prevent and Control Overweight, Obesity and Diabetes (ENPCSOD) and have become opportunities for private entities to be “part of the solution” to addressing the obesity problem.^14,29^ As such, PPPs between the transnational F&BI (hereafter F&BI or “the private partner”) and different government agencies have been prevalent in Mexico. While some PPPs with the F&BI have existed for a long time, others have emerged in response to the government’s strategies and policies aimed at tackling obesity,^30^ such as an industry-led front-of-pack nutrition labelling policy. This study analyses how four PPPs between the Mexican government and the F&BI and allies are working to achieve their goals. We focused on four PPPs that aimed to control obesity, increase physical activity levels, and improve access to drinking water and sanitation. We critically assess these four PPPs’ objectives, scope, reported impacts, governance principles and perceived risks and benefits.

## Methods

 This is an exploratory study using a documentary analysis and interviews of four PPPs in Mexico. We selected four PPPs (listed in Box 1) purposely following a series of criteria described in Box 1 and, after conducting a literature review.^31^ The selected PPPs aimed to provide a service or a program in the country, functioning as partnerships in policy implementation targeted at vulnerable groups, and include children and low-income populations. The cases included partnerships either that started while or after ENPCSOD was negotiated (2011-2012) and until 2019, when we started this study.

**Box 1. Inclusion Criteria of Public-Private Partnerships Selected for Analysis**

**Inclusion Criteria**

a. Participation of a private partner that is a transnational food or beverage corporation based in Mexico.

b. An explicit aim to provide or improve a nutrition and health service (obesity prevention, physical activity, nutrition education, water sanitation) to vulnerable populations such as children and/or communities living in poverty.

c. Was established or has been operating for at least 6 years, as having emerged during or after the launch of the ENPCSOD (2013), at a time when industry increasingly wanted to show themselves as part of the solution to the obesity epidemic, and when Peña Nieto’s government agenda included a plan to combat the obesity and diabetes epidemic.

d. Existence of sufficient documentation (reached by consensus of three investigators) to allow a thorough analysis from the perspective of both the private and the public partners.

e. Has a substantial number of beneficiaries and breadth of scope. This is defined as >5000 beneficiaries per program in the selected timeframe of analysis (2 to 5 years).

**PPPs Selected**

a. “*Centros de Hidratación*” program lead by Escuelas Sustentables A.C., an NGO sponsored nationally by Coca-Cola and in collaboration with the Secretariat of Education, mainly through INIFED.

b. “*Agua Saneamiento y Salud*” [Water Sanitation and Hygiene] project promoted nation-wide, by Pepsi-Co, the IDB and the Mexican water agency CONAGUA.

c. “*Ponte al 100*” launched by Coca-Cola with the Secretariat of Health and the National Commission of Physical Culture and Sports.

d. “*Nestlé por niños saludables*” (previously “*Nutrir*”) [Nestlé for healthier kids] and the local Secretariat of Education and Secretariat of Health.

-------------------

Abbreviations: PPPs, public-private partnerships; ENPCSOD, National Strategy to Prevent and Control Overweight, Obesity and Diabetes; NGO, non-governmental organisation; INIFED, Instituto Nacional de Infraestructura Física Educativa; CONAGUA, Comisión Nacional del Agua; IDB, Inter-American Development Bank.

We critically examined these PPPs by analysing public documents and conducting key informant interviews to compare the information provided by the private and public partners for each program. First, we used the Logical Framework Approach (LFA), a methodology mainly used for designing, monitoring and evaluating projects, to understand the goals, objectives, outputs, and activities (implementation) of each PPP.^32^ We also applied this approach to understand each program’s indicators for monitoring their implementation and evaluating their impact,^32^ as the LFA helps to articulate a common interpretation of the objectives of a project and provides a clear basis for monitoring progress and verifying achieved objectives. While we did not aim to map all the components of each PPP in our study, we used the LFA to identify the clarity and transparency of their objectives, outputs, and activities.

 Secondly, we assessed the governance principles followed by the partners in arranging and executing the PPPs. We used the principles of good governance previously described in the literature^25,33,34^ to assess each PPP. These principles have consistently been used to assess health programs and interventions by intergovernmental bodies and public health research.^34^ For this study, we determined that gaps in any of the information given by the documents and interviewees might imply weaknesses of the governance principles. Finally, we analysed the perceived risks and benefits of each PPP, the actors involved, and their relevance to the nutrition policy agenda.

###  Data Selection

 We conducted a document review related to the selected PPPs and the Mexican context (documents published between 2012 and 2019). We first performed an initial search between February and March 2020 using Google and Google Scholar with key search terms outlined in Box 2. We then used the snowballing technique and conducted a forward search for documents that cited previously identified sources. We also made 50 freedom of information (FOI) requests to different national and sub-national government institutions involved in the PPPs of study. Of these, 22 official documents were obtained. We contacted the Secretaría de Salud (SSA), Secretaría de Educación Pública (SEP) and the Instituto Nacional de Infraestructura Física Educativa (INIFED). We also contacted the local subsidiaries within Mexican states where the PPPs operated and were promoted. Overall, a total of 170 documents were identified for our analysis, including industry annual reports, civil society group websites, press releases or reports, journalistic notes, and FOI official documents.

**Box 2. Key Terms Used for Systematic Searches by Public-Private Partnership Studied**

**1. Centros de Hidratación**

Search terms: “Centros de Hidratación,” “Escuelas Sustentables,” “Coca-Cola,” “bebederos,” and “escuelas”

Webpages: Google, Gobierno de México, inifed.gob.mx, escuelas-sustentables.org.mx, coca-colamexico.com.mx, fundaciococacola.com

**2. Agua Saneamiento y Salud**

Search terms: Agua, saneamiento, salud, “PepsiCo,” Pepsi, Conagua, “Comisión Nacional del Agua,” “de la fuente a la casa,” “Banco Interamericano de Desarrollo,” and “BID”

Webpage: Google, Gobierno de México, iadb.org, pepsico.com.mx

**3. Ponte al 100**

Search terms: “Ponte al 100,” “Ponte al cien,” and “Programa Nacional de activación física Ponte al 100”

Webpage: Google, Gobierno de México, fundacionmovimientoessalud.org.mx, and capacidadfuncional.com.mx

**4. Nestlé por niños saludables**

Search terms: “Nestlé por niños saludables,” “Nutrir,” and “Nestlé”

Webpage: Google, Gobierno de México, nestle.com.mx, Gobierno Estado de México, Veracruz, Ciudad de México, Guanajuato, and Michoacán

The first and second authors conducted 25 semi-structured interviews with purposively selected key informants from March 2020 to June 2020, after inviting 41 people identified through the documentary review and using the snowballing technique. The sample included a variety of actors: seven experts on PPPs (academic or international organisations), eight operational personnel or consultants for the PPPs studied, five civil society members or foundations’ representatives, two private sector representatives, and three government representatives familiar with the PPPs included in our study. The interviews were conducted during the Covid pandemic, so several interviewees were either not available to participate (n = 4), did not respond (n = 9), or declined to participate (n = 2). Saturation was reached when similar themes emerged. The interviews lasted between 40 to 60 minutes and followed a semi-structured interview guide covering the origin of the PPP, their purpose, operations, evaluation and monitoring, brand and company associations, and perceived benefits and weaknesses (Supplementary file 1). The guide was informed by selected literature on good governance and the LFA (Supplementary file 1).

###  Analysis and Synthesis

 The interviews were transcribed verbatim, and the transcriptions and documents selected were analysed thematically. The research team followed an initial coding framework based on our research questions and informed by the select literature on good governance and the LFA (Supplementary file 1). The framework was discussed and further refined by the team in an iterative process. AC and EO coded five transcripts, after which the framework was adapted and applied to all transcripts and documents using NVivo 11. Codes were checked between the researchers to ensure accuracy and coherence. Two co-authors (AC and EO) reviewed 10% of the codes for consistency and reliability purposes (>0.75 kappa value). Authors held regular discussions, which allowed reflexivity and discussion of potential biases on the analysis due to coders’ role in the topic, familiarity, and beliefs about it. The analysis was conducted between June and December 2020.

 The theoretical perspective was underpinned by a “realist” or “contextualist”^35^ view of the data as representing individuals’ intersubjective meanings within a socially and materially structured (real) environment. As a result, we cannot fully know the “truth” of a situation, but we can get closer to it through methods such as listening to and triangulating the views of people who experienced it.^36^

 We present our qualitative synthesis by topic themes grouped into (*a*) the programmatic aspects of the PPPs, based on the LFA components used for the interviews and coding, and (*b*) principles of good governance (described in Box 3) in the PPPs.

**Box 3. Definitions of Good Governance Principles Used for the Analysis**

**Accountability:** Decision-makers in government, the private sector and civil society organizations involved in health are liable to the public, as well as to institutional stakeholders. This accountability differs depending on the organization and whether the decision is internal or external to an organization.^34^

**Credibility:** It includes trust and trustworthiness. Trustworthiness relies on the institution and on the perception the public has of the institution.^37^

**Efficiency and effectiveness:** Processes and institutions should produce results that meet population needs and influence health outcomes while making the best use of resources.^34^

**Ethics:** The commonly accepted principles of PPPs include beneficence (should lead to public health gain); non-maleficence (must not lead to ill-health); autonomy (should not undermine each partner’s autonomy); and equity (benefits should be distributed to those most in need.

**Fairness:** is characterized by equity, respect, justice and stewardship of the shared goods, both among people and in their relations to other living beings.

**Integrity:** Integrity exists when there is consistency “among what an institution does, what it says it does and what it is obliged to do”^37^ (p. 115). Consistency is needed between the practices, its mission and its purpose.

**Participation:** All men and women should have a voice in decision-making for health, either directly or through legitimate intermediate institutions that represent their interests. Such broad participation is built on freedom of association and speech, as well as capacities to participate constructively. Good governance of the health system mediates differing interests to reach a broad consensus on what is in the best interests of the group and, where possible, on health policies and procedures.^34^

**Stewardship:** It is defined as the function of the government responsible for the welfare of the population and concerned about the trust and legitimacy with which its activities are viewed by the citizenry.^22,38,39^

**Transparency:** Transparency is built on the free flow of information for all health matters. Processes, institutions and information should be directly accessible to those concerned with them, and enough information is provided to understand and monitor a health matter.^34^

**Conflict of interests:** CoI arises in circumstances where there is potential for a secondary interest (a vested interest in the outcome – of the programs) to unduly influence, or where it may be reasonably perceived to unduly influence, either the independence or objectivity of. Professional judgment or actions regarding a primary interest (in this case the program to be delivered).^40^

----------------

Abbreviations: CoI, conflict of interest; PPPs, public-private partnerships.

## Results

 The details of the four PPPs studied are presented in Table 1. This includes the partners involved, the geographical area they operate in, the name of the program given by the private and the public partners (which can differ), and the project aims, beneficiaries, and their potential impact.

**Table 1:** PPPs Studied, the Private and Public Actors Involved, the Programs Related to Them and the Aims

**Programa Centros de Hidratación/Programa Nacional de Bebederos Escolares (2011-2018)**	
Partners	Arca Continental, Coca-Cola México, Coca-Cola FEMSA, Escuelas Sustentables A.C., Fundación Bepensa, Fundación Coca-Cola, State school construction institutes (INIFED local authorities), and Secretariat of Education (SEP local authorities).
Geographical Areas	Several states throughout the country including Ciudad de México, Coahuila, Durango, Guanajuato, Jalisco, Nuevo León, and Quintana Roo.
Objectives	***Programa Centros de Hidratación*** To provide safe and potable water to schools by installing water fountains in pre-schools, primary and secondary schools, mainly in rural and indigenous communities, helping to improve health of students, teachers and community members. The priority has been in the poorest areas of the country. ***Programa Nacional de Bebederos***** SEP/INIFED ** (Part of the Education Reform before 2016 as part of “Escuelas Dignas” and after 2016 as “Escuelas al CIEN” executed and evaluated by INIFED)One of the specific objectives of the national Educational Reform is to install and maintain school drinking fountain systems that provide a continuous supply of drinking water in said schools, in accordance with the provisions issued by the INIFED.
Beneficiaries and impact	*Programa Centros de Hidratación* has installed 1711 water fountains (*Escuelas Sustentables* website). According to INIFED they installed 13 326 water fountain systems where 2000 were provided by “alternative sources of funding” (INIFED accountability report 2012-2018).
Evaluation	Only some reports by INIFED were available. Some numbers are provided.
**Agua Saneamiento y Salud (2012-2020)**	
Partners	CONAGUA, IDB, and PepsiCo.
Geographic area	Pilot project: initially they identified rural communities in 4 municipalities in Estado de México, Michoacán, San Luis Potosí and Veracruz. We did not find information about other entities.
Objectives	PRODI– CONAGUA/IDB The objective is to seek development schemes aimed at providing drinking water to dispersed populations.
Beneficiaries and impact	UD$ 5 million from PepsiCo for 850 000 beneficiaries (in total until 2025) initially. Between 2011-2016 – US$ 7 million for 772 181 beneficiaries.
Evaluation	Yes, bi-annual reports are available in IDB page, nevertheless a report on the impact of the program is not available neither in the IDB portal nor provided when filing a FOI request to CONAGUA.
**Ponte al 100 (2013- 2020)**	
Partners	CONADE, CONDEBA, Secretariat of Health, Secretariat of Education (national and sub-national level), Industria Mexicana Coca-Cola, Fundación Coca-Cola, Fundación Movimiento es Salud A.C., Federación Mexicana de Medicina del Deporte, Fundación Azteca, Fundación Carlos Slim, Instituto Mexicano del Seguro Social, Instituto de Salud Pública de la Universidad Anáhuac, Instituto Politécnico Nacional, MOVISA, Policía Federal, Tecnológico Nacional de México, and The Aspen Insitute.
Geographical area	Estado de México for the first 3 years with 105 schools, and later many others. Operation in 29 states by 2014 (SEP Progress Report, September 1, 2015) reducing its scope to 22 states in 2018.
Objective	The objective of *Ponte al 100* is to guide the general population through three main paths: measure how the body is doing (diagnoses), evaluate how fit a person is, and prescribe exercise and a diet individually to change habits.
Beneficiaries and impact	According to Coca-Cola, more than 4 million diagnoses have been conducted to more than 2.6 million people. According to Coca-Cola 900 000 of 4700 schools have been attended through the program. According to the *Fundación Movimiento es Salud A.C.* website, they have measured 2 569 393 boys and 2 601 032 girls.
Evaluation	Yes, a performance evaluation by CONEVAL and a process evaluation done by consultants for CONADE on the “*Programa de Cultura Física.*” Also, CONADE and the General Directorate of Policy Evaluation of SEP conducted a diagnosis report of the “*Programa S269 de Cultura Física y Deporte.*”
**Nestlé por niños saludables (2006- 2020)**	
Partners	DIF (local authorities), Nestlé, SEP (local authorities), and SSA.
Geographical area	Estado de México, Guanajuato, Mexico City, and Veracruz.
Objectives	*Nestlé por Niños Saludables* (Nestlé for Healthier Kids) seeks, to promote the adoption of healthy living habits with children from 0 to 12 years of age through several programs and tools developed by the company(Nestlé Report 2016-2018).
Beneficiaries and impact	Every year they train 650 000 children and 6000 schools and 1 million parents. According to Nestlé they have reached 5 million children, 60 thousand parents and 10 000 teachers in 6000 schools (Nestlé website: https://www.nestle.com.mx/csv/iniciativas_globales/nxns; consulted February 11, 2020).
Evaluation	Qualitative study by the private partner evaluating the change in knowledge after the intervention. No information provided through the FOI requests in the states where the program operates.

Abbreviations: PPPs, public-private partnerships; INIFED, Instituto Nacional de Infraestructura Física Educativa; SEP, Secretaría de Educación Pública; PRODI, Programa de Desarrollo Integral para Organismos Operadores de Agua Potable y Saneamiento; IDB, Inter-American Development Bank; CONAGUA, Comisión Nacional del Agua; CONADE, National Commission for Physical Culture and Sport; MOVISA, Movimiento por una Vida Saludable; DIF, Desarrollo Integral de la Familia; SSA, Secretariat of Health; CONDEBA, Consejo Nacional para el Desarrollo de la Educación Física y el Deporte en la Educación Básic; CONEVAL, Consejo Nacional para la Evaluación de la Política de Desarrollo Social.

 Our findings suggest that the main instruments used to consolidate the partnerships were written agreements between the government (either with local or federal authorities) and an organisation sponsored by the F&BI or directly with the company’s philanthropic foundation. Three partnerships were announced in public gatherings by local or federal government officials and outlined in corporate reports, but we could not find accurate information available on the engagement from government FOI replies.

###  Types of Public-Private Partnerships Identified

 We identified three types of arrangements between the public and private sectors. The first type is when the private sector joins a public action (program), typically only for a specific part or during the implementation phase, which is often restricted to donations (eg, *Centros de Hidratación* and *Ponte al 100*). The second one identified was when the private sector presents its own program and the public partner had little influence on its design but had the freedom to implement it, either to complement a program already running or to start a new one (eg, *Agua Saneamiento y Salud*). The third type was when the private sector leads, designs, implements and acts as the decision-maker of the PPP. While the government provides access to the venues, the private partner implements the program in various settings, such as community centres, municipality government offices, schools, or community centres (eg, *Nestlé por niños saludables* and *Centros de Hidratación)*.

###  Programs’ Aims, Objectives, Design, and Implementation 

 When studying the aims, objectives, design, and implementation of the four PPPs studied we found: (*i*) in some cases there were key discrepancies between the aims described by different partners involved (either documents or verbally described by interviewees); (*ii*) uncertainty about the delivered actions, scope of the intervention and benefits to the population; (*iii*) unclear information about the indicators, or existing evaluations of the PPPs and their overall impact on health outcomes. Table 2 provides examples of how different sources describe the objectives, actions, indicators, goals and (or) impact of the PPPs.

**Table 2:** Examples of Objectives, Actions and Indicators Described by the Private Partner (or Through the Front Groups) and by the Public Partner in the Various Case Studies

**Domain**	**PPP Example**	**Private Partners**	**Public Partners**
Objectives	*Ponte al 100 *	*“Ponte al 100 evaluates people’s physical state, analyzes it, and prescribes an appropriate diet and exercise regime” (*Movimiento es Salud Foundation website). *“*Ponte al 100* is an educational initiative to empower children, young people, teachers, and families to take responsibility for their physical health, structuring the school community as the axis of action, and incorporating physical exercise, nutrition, and school infrastructure” (*Movimiento es Salud website). *“We did a pilot plan in the State of Mexico trying to identify how we could evaluate the effect of physical education in primary and secondary schools. We did a pilot plan to see if we could establish a set of physical tests with indicators for Mexicans […] Ponte al 100 is basically the set of tests” *(Movimiento es salud representative).	*“The objective of the *Ponte al 100* pilot program was to help fight sedentary lifestyles and lower rates of overweight and obesity, by engaging in physical activity and adopting personalized nutritional recommendations made available to the population based on Functional Capacity Assessments (*Ponte al 100*), and encourage people to play an active role in preserving their health” *(CONADE Reply to Senators, December 6, 2017).
	*Nestlé por niños saludables*	*“The program was created as a social responsibility strategy […] through this program, basic nutrition knowledge could be instilled to help children establish healthy lifestyles […] Our objective was to be the teachers’ ally and provide them with training and materials[…]” *(Private partner interviewee).	*“With the purpose of preventing diseases by promoting good eating habits, the Secretariat of Education of Veracruz (SEV), in coordination with the company Nestlé and the Gastronomic Council of Veracruz, signed a collaboration agreement for the ‘Nestlé por Niños Saludables’ program” *(Veracruz Government Newsletter, March 12, 2019). *“Because obesity is a complex problem, it is important to establish joint actions, as is the case of ‘*Unidos por Niños Saludables*,’ which today joins this cause and will undoubtedly have important results in changing children's habits, she said [the Secretary of Health Mercedes Juan]” *(Secretariat of Health press release, August 28, 2014).
Actions	*Centros de Hidratación*	*“In collaboration with the non-profit *Escuelas Sustentables*, we continue expanding the Hydration Centers program with the aim of supplying communities with safe drinking water and promoting responsible use of water among young people” *(Mexican Coca-Cola Industry Sustainability Report, 2015 p. 21).	*“Article 11 of the General Law on Educational Physical Infrastructure states that the existence of sufficient drinking water fountains with a continuous supply of drinking water will be guaranteed on all premises for school use in accordance with the guidelines issued by the Secretariat of Health in coordination with the Secretariat of Public Education” *(General guidelines for drinking water fountains, DOF 12/23/2015). *“Nutrir, Unidos por Niños Saludables promotes nutritional knowledge in school age children through nutritional orientation sessions with options for games and recreational activities which can be implemented in classrooms, cafeterias, community centers, or at home in a progressive and flexible manner” *(Guanajuato Government Bulletin, December 10, 2017).
	*Nestlé por niños saludables*	*“Through partnerships with NGOs and local secretariats of education, we provide material for schools and train teachers to offer nutritional orientation sessions” *(Nestlé Report 2016-2018).	
Indicators used	*Centros de Hidratación*	*Water fountains installed *(Fundación Escuelas Sustentables A.C. and Mexican Coca-Cola Industry Sustainability Report, 2016 p. 22).	*“Number of schools with water fountains (regardless being working or not) by the end of the assessment period (INIFED) and number of water fountains installed”*(INIFED accountability report 2012-2018).
	*Ponte al 100*	*Overall number of person’s “functional capacity” tests performed *(Mexican Coca-Cola Industry Sustainability Report, 2014 p. 19).	*“Same as the private entity but with no mention of the education goals or impact of the program” *(CONADE Reply to Senators, December 6, 2017).
Goals and/or impact	*Nestlé por niños saludables*	*“We have impacted 2.3 million children and more than 1,000 teachers in more than 6,000 schools nationwide” *(Nestlé Report 2016-2018). *“For 13 years, the program “Nestlé por Niños Saludables” has been implemented successfully in different parts of the country, impacting more than 4.5 million children, in more than 6,000 public and private schools throughout Mexico, with a special focus on Mexico City, the State of Mexico, Guanajuato, and Veracruz” *(Nestlé Press Release, March 14, 2019).	* “The Program “Nestlé por Niños Saludables” will benefit 650 thousand children and young people at 6 thousand schools in the state, federal and state schools, from preschool to junior high, and will also serve indigenous and special education schools” *(Veracruz Government Newsletter, March 12, 2019).
	*Agua, saneamiento y salud*	*“As the first private sector donor to contribute to the IDB fiduciary fund in 2011, the PepsiCo Foundation’s 7 million dollar investment in the AquaFund (made between 2011 and 2016) helped catalyze 547 million dollars in total funds through April 2019 and provided new or improved access to safe water and sanitary services to more than 765 thousand people” *(PepsiCo Sustainability Report 2018).	*“The Program for Sustainability of Drinking Water and Water Treatment Services in Rural Communities IV was financed by means of a 450 million dollar IDB loan to the Mexican government, which will eventually benefit 600 thousand people with new access to drinking water, and around 390 thousand people with new access to sewerage and basic water treatment in rural zones in 31 states of Mexico” *(IDB Press Release, 2014).

Abbreviations: PPP, public-private partnership; CONADE, National Commission for Physical Culture and Sport; NGOs, non-governmental organisations; INIFED, Instituto Nacional de Infraestructura Física Educativa; IDB, Inter-American Development Bank.

 For the program Ponte al 100, different objectives were reported by each partner (See Table 2). While Fundación Movimiento es Salud A.C., the association funded by Coca-Cola aimed to assess the “physical capacity—without a clear definition of what that means—of schoolchildren,” the government stated it was a program “to improve physical activity among schoolchildren and their families.”

 The program Ponte al 100 started in 2013 as a pilot in the State of Mexico and it became part of the national program of physical activity and the National Commission for Physical Culture and Sport (CONADE), the SSA and Coca-Cola. As one informant described: “*Ponte al 100 is basically the set of tests to measure physical activity capacity” *(Civil society representative), and confirmed that the aim of the program was adapted to fulfil the request of the SSA to enable the partnership at the federal level:“*We changed the original objective [note: participant did not provide information on original objective] and converted it into a physical activity program, aiming to make people healthier. I had the first permits from the SSA [to operate in schools at the time]. We sought authorisation with Mercedes Juan [the Secretariat of Health]. Pablo Kuri [the Under Secretary of Health] didn’t like the program because we were not a [obesity] preventing program as it is strictly defined in the NOM-008 for obesity prevention” *(Civil society representative).

 The General Law on Educational Physical Infrastructure proposed “*to install drinking water fountains in public schools, from preschool to high school*” (La Jornada, November 13, 2013). According to the law, the aim was “to benefit 242 621 schools reaching 30 115 977 students in the national education system,” where the “private partner will support the instalment of the fountains.”In 2015, INIFED was appointed to provide schools with water fountains as part of the “Programa de Reforma Educativa” following the approval of the law. It was a program aiming to improve the schools’ infrastructure and, overall, the education system in the country. Escuelas Sustentables A.C., funded by Fundación Coca-Cola, had a slightly different aim, which was to provide drinking water to communities and promote responsible use of water. In the agreements we reviewed (obtained through FOI requests) Escuelas Sustentables A.C. agreed to provide support to install and evaluate the water quality and access to clean water. Also, it committed to providing guidance and training to manage and maintain the water fountains and requested the government that for each water fountain it installed, the government should install one with public funds. The agreements also mentioned that the F&BI (and not the foundation) be acknowledged for this work; hence the installation would carry the name of a soda company, a form of branding beneficial to the industry:

 For the program *Nestlé por niños saludables*, the public partner framed the PPP as “promoting knowledge in school-aged children,” while Nestlé, the private partner, reported that the main aim was to develop material and make it available to teachers to promote healthy habits among schoolchildren (Table 2). This limits the private partner intervention in the program to facilitate educational materials, which the public partner must deliver to teachers and students.

###  Uncertainty About the Actions, Scope, and Benefits of the Intervention

####  Information Management and Implementation

 Information management was unclear for some of the PPPs. Private partners reported having records and results but mentioned they were not publicly available or were not shared with our research team. For the public entity, operating rules of public programs exist, but some of them were written a posteriori,^41-44^ were not appropriately followed,^45^ or in some cases, as some FOI responses indicated, were non-existent. Some indicated that another authority was responsible for it, but none of the submitted FOIs yielded relevant information despite the requests being made to various institutions at national and local levels. Some public entities either delayed or denied answering us. In some cases, they pointed out that it was the responsibility of the private sector, as shown in Table 3, without it being. The fact that the private sector held records of the program’s implementation, beneficiaries records, and details on indicators of impact was not perceived as a conflicting issue.

**Table 3:** Examples of FOI Answers for Centros de Hidratación and the Agreements With Fundación Coca-Cola

**Text on the FOI Request**	**FOI Request Delayed**	**FOI Request Denied**
The description of “Escuelas Sustentables” in the agreements says: “It has an agreement with Fundación Coca-Cola AC who is the sole sponsor for the donation of the Coca-Cola Hydration Centers. The agreement is intended to arrange collaboration, participation, and execution of the installation of Coca-Cola Hydration Centers in public educational establishments” (Agreement for the donation and installation of 26 Coca-Cola Hydration Centers, INIFED Coahuila, April 2014).	The information requested is in print format, so it is not in digital format, 31 contracts concluded in 2014, 2015, and 2016 are derived, the documentation is integrated in 38 folders with a volume of more than 8600 sheets (Answer to FOI requests from INIFED Coahuila, May 2020).	In accordance with the provisions of Article 131 of the General Law of Transparency and Access to Public Information, a search was carried out in the files […] and it is not in the power of this entity, nor is there any precedent of the subject referred to in the INIFED. Due to the above, and since it is a matter that belongs solely to the private initiative, it is suggested to submit to the consideration to the companies involved (Answer to FOI request from INIFED, October 2020).

Abbreviations: INIFED, Instituto Nacional de Infraestructura Física Educativa; FOI, freedom of information.

####  Program Indicators, Monitoring, and Evaluations

 The studied PPPs’ indicators for activities, outputs, or programs’ impact were not clear in the private or public partners’ documents. For instance, *Ponte al 100* provided information on how many “functional capacity assessments” were conducted (outputs of the intervention). Coca-Cola’s 2015 annual report mentions that *Ponte al 100* performed personalised diagnoses on 1.6 million Mexicans, and in 2016, on 2.6 million people. But CONADE reported a total of 1 364 891 “functional capacity” assessments during the operation of the program from 2013 to 2015 (CONADE Reply to Senators, December 6, 2017, and FOI given by CONADE, October 2020). Nevertheless, it did not provide how many or, if at all, people changed their physical activity patterns or improved their diet (outcome).

 For *Nestlé por Niños Saludables*, we found similar issues, with interviews from the private partner mentioning there were in-house reports on the progress of the program, and a press release by Nestlé stating that “The program has shown that children have increased their consumption of vegetables and fruits, in addition to increasing their physical activity,*”* but with no specific data or ways to verify of such claims.^46^

 Likewise*, Centros de Hidratación* outlined indicators to measure progress, with the number of water fountains installed, but it was unclear how these changed the drinking patterns for children and therefore improved health (the impact the national program claimed to be aiming for).

 For *Agua Saneamiento y Salud*, PepsiCo reported that their donations to the Inter-American Development Bank (IDB) provided new or improved access to safe water and sanitary services to more than 765 000 people (PepsiCo Sustainability Report 2018), while the IDB provided the “number of homes with improved access to drinking water as 4 000 000 in 2018” (IDB Press Release, 2014) and to have reached a total of 772 181 persons (Table 1).

####  Impact and Effectiveness of the Programs

 In terms of evaluation and effectiveness (as defined in Box 3) of the program, we found that there were three evaluations done, one each for *Centros de Hidratación*, *Ponte al 100* and *Agua Saneamiento y Salud*. Despite all of them being from external evaluators, there were inconsistencies with the results reported by the private sector. For example, while the private sector entities reported on achievements (impact), the public sector reported on the goals, not on the impact of the interventions. None of the four PPPs studied had public access to complete information on the impact evaluations of the programs.

###  Governance

 The principles of good governance as defined for this paper (See Box 3) were not reflected in the information gathered from the programs, including those of accountability, transparency, fairness, participation, integrity, and credibility.

####  Accountability, Transparency, and Credibility

 Considering the areas of interaction, confrontation, and synergies between public and private actors, we found that public figures with political ambitions, such as governors, communicated openly about their interactions with soda companies or their subsidiaries. In the same way, the soda companies framed the cooperation with local governments and their common social goals as part of their “corporate social responsibility.” For example, a Coca-Cola report states, “*The Coca-Cola Foundation joined forces with the CONADE and the SEP for the implementation of ‘Ponte al 100’” *(Informe de sustentabilidad, Industria Mexicana de Coca-Cola, 2014 p. 19).

 However, the clarity of the private sector involvement became less clear in the most recent corporate reports reviewed,^47-52^ which describe the PPPs as part of their corporate social responsability actions with a glimpse of information on their web pages and charitable branches. At the federal level, reports do exist but leave most of the information, indicators, results, and impact quite unclear, as described above, calling into question transparency, accountability, and stewardship issues.

 Some confrontations between the public and private actors were perceived through the history of each PPP studied. For *Ponte al 100*, the discussion about accountability generated a clear conflict for the program and for the PPP. In September 2016, Senator Martha Tagle Martínez filed a petition to CONADE for getting information about the details of the program, including all the names of the governmental and non-governmental organisations (NGOs) and the contribution of each one to the program. She presented the budget assigned to this program in 2013 and in 2014 and questioned how this was spent, arguing that “an official registration of the program is inexistent,” the program “left financial damage while Mena was the head of the CONADE,” and that “some of the problems this program had were discussed in the Semanario Proceso” where “accounts about the delay and the lack of transparency is investigated.” The senator pointed to the discrepancy about the participation of Coca-Cola in providing materials and equipment for the execution of the program. The senator also discussed the expenditure of money, on the open tenders, duplicated expenses, and doubts about corruption around the program’s finances. Additionally, compared to its launch, when the PPP was applauded by many politicians, by the end of it, the PPP was highly criticised by many scholars because of its ineffectiveness in reducing childhood obesity.^53^

 We learned that the program *Nestlé por Niños Saludables *will continue in the State of Veracruz until 2024. Also, Fundación Movimiento es Salud was still operating *Ponte al 100* as of November 2020, when we conducted the study, but as a program called Prospectiva2030 in 5000 schools. We obtained one 2018’s agreement, signed by the State of Campeche public authorities, stating that the program aimed to “follow up the schools for three years.” Only one government official mentioned this information and clarified that although they were trying to stop this partnership in the State of Yucatán, it was still running in other states, and it was up to the local authorities to accept it. In the case of *Agua Saneamiento y Salud*, the involvement of the soda industry is not obvious and has not been recognised in any document, a clear issue with transparency. For example, for *Agua Saneamiento y Salud*, none of the documents mentioned any potential “risks” given the conflict of interest (CoI) that promoting and managing access to drinking water to small populations implies for soda companies. Indeed, some community activists pointed out that the PPP is strategically executed in places where the same soda companies have their bottling plants and added* that *“a bilateral organisation carries out the management of the program, so the visibility of the participation of the private sector is diluted” (member of civil society).

####  Ethics, Integrity, Participation, Fairness, and Stewardship

 The PPPs studied were unclear on their ethical principles, such as involving no harming principles, being directed to people, and respecting autonomy and equity. Although NGOs financed by the private sector declared corporate values and missions on their webpages, principles to achieve these mission statements in the four PPPs were not established in the accessed agreements or evident in their execution, as reported by our interviewees.

 Integrity (as defined in Box 3) was also at stake in some cases. A clear example of this discrepancy was found through the FOI requests for the program *Nestlé por Niños Saludables* in the State of Mexico. The private entity mentioned that “*For 13 years, Nestlé por Niños Saludables has been successfully implemented in different parts of the country, impacting more than 4.5 million children, in more than 6000 public and private schools in the country with a special focus on Mexico City, State of Mexico, Guanajuato and Veracruz*” (Press release by Nestlé, March 14, 2019). Meanwhile, the public entity, the education authority of the State of Mexico, replied to our FOI request, saying that “after a meticulous search in the archives of this Administrative Unit, these documents are not available”* (*Response of the Secretariat of Education of the State of Mexico, 2020*). *

 Both *Nestlé por Niños Saludables *and *Ponte al 100* were granted access to children’s records (including the personal information of thousands of children and students). Data protection of participants was not mentioned in any of the agreements reviewed or in any of the interviews conducted, but the PPP agreement mentioned that Fundación Movimiento es Salud A.C. had to keep data confidential.

 As with the principles of stewardship, fairness, transparency and accountability, we found that each of these programs has been subject to criticism. Some Congressmen or public-interested groups through the media made strong calls for better accountability.^53,54^

####  Conflicts of Interest

 CoIs are not part of the good governance principles as defined in the existing literature, but represent a key issue encountered in PPPs, particularly the institutional CoI involved in such arrangements. According to the definition of CoI we used (See Box 3), in all PPPs, the CoI for the public partner was inherent. The transnational F&BI involved in the PPP’s core aim was to improve their profits by selling their products. The portfolios of products of the F&BI participating in the studied PPPs were mainly composed of ultra-processed foods or beverages high in sugar, salt or fat. Thus, their primary activity went against the government’s duty to protect and promote public health.

 The public partner’s employees in the PPPs, both those working in the field directly with beneficiaries and those at the coordination level, were largely unaware that the programs had private partners or that the public sector made commitments to the private sector.^37^ For example, for *Centros de Hidratación*, an interviewee mentioned, “*The only one who knew who the sponsor was, was the school director, and he decided if it had to be known by the parents or not.*”

 Some of the interviewees did not consider PPPs with F&BI to be a CoI. However, some of the agreements include clauses requiring the public sector to mention, acknowledge, or label program material with trademarks. For example, this included naming Coca-Cola on a silver plate next to the water fountains or using Nestlé logos on the material provided. Likewise, implementers mentioned that brand promotion at the sites where the programs were implemented was present either in a subtle (eg, brand colours) or in an obvious way (eg, logos), but neither the interviewees nor the beneficiaries considered them as relevant or inappropriate.

 Some of the public actors interviewed did find it difficult to understand and to accept industry collaborations, and they mentioned that they have tried to change the behaviour of local authorities around CoI, but sometimes, further up in the power chain of actors, relationships with the F&BI prevail and are “untouchable.”

####  Perceived Risks and Benefits 

 Some experts and implementers interviewed perceived risks associated with PPPs because of the potential lack of continuity of the programs and their “negative impact on public health” (Expert). One expert noted that there was a lack of understanding among public servants that philanthropic organisations involved in PPPs are in fact third parties for the F&BI. As an expert noted:

 “*On multisectoral governance, it looks great to involve civil society; the problem is what kind of civil society participates because when you see in detail, they are NGOs sponsored by the F*&*BI”* (Expert).

 Other risks associated with these PPPs were related to tax deductions for philanthropic foundations, as an expert suggested:

 “*The tax exemption generates a structure to create a bureaucracy for tax deductions. The moment it [the tax exemption] is given to the private [partner], they are no longer public resources [and], a problem of accountability emerges, it [the private partner] is not being held accountable, or we ignore if children [beneficiaries] improved [their habits] or not” *(Expert).

 Despite some of the risks and limitations of programs executed by the government in collaboration with the F&BI, as described by a public official, there was “the sense of a financial relief for a stretched [public entity].” Nevertheless, we found that public officials were less inclined to recognise that interactions with the private sector might influence their own institutions’ policies and practices. As mentioned by an interviewee:

 “*In Monterrey, Fundación FEMSA work on the water looks great because they’re from Monterrey, and it’s almost a source of pride, and they have a good water system... Instead, Coca-Cola in San Cristóbal had to negotiate a lot; they were perceived as the bad ones” *(IDB informant).

 Likewise, the technological and practical knowledge of the private partners was also seen as a benefit because “they know what they are doing in the field” (civil society organization) and as mentioned by one informant: “disclosing the relationship with the private partner [to beneficiaries] is not relevant” (government official), arguing that having corporate support is better because “otherwise it wouldn’t be provided.”

## Discussion

 In this study we analysed how four PPPs between the Mexican government and the F&BI and allies are working to achieve their goals. We focused on four PPPs that aimed to control obesity, increase physical activity levels, and improve access to drinking water and sanitation. We critically assess their objectives, scope, reported impacts, governance principles and perceived risks and benefits. We found that among the PPPs studied, there has been a lack of synergy between the goals, aims and actions of the public and private partners involved in delivering the intended benefits through their programs. The aims diverged as there was no clear information on the execution of the programs conducted under the PPPs and little details about their results or impact on public health outcomes. Some of these programs have been evaluated by external parties, but the results did not coincide with the initial aims or goals originally established, therefore making it impossible to understand if these PPPs were effective in contributing to the public health agenda of preventing NCDs. We found issues with accountability, transparency, and credibility, as well as ethics, integrity, participation, fairness, and stewardship, a finding that is aligned with what other authors have previously found and discussed in the literature.^25,37,55,56^

 This study adds to evidence that PPPs raise concerns about how effective they are in contributing to the public health agenda (framing effects, influencing the agenda, reporting results, etc). Although PPPs in Mexico might sound commendable, there is little evidence that the partnerships achieved what policy-makers hoped and what the local populations might have expected. The efforts to address obesity or access to water, through tailored programs run by PPP arrangements studied here have failed to show an effect on the population’s health, mainly because there are no, to our knowledge, clear indicators or impact evaluations available. If there are indicators, they were not consistent within partners or have been found to be inefficient and poorly executed. In the past couple of years, scholars and activists have suggested mechanisms for accountability, ethics and transparency on PPPs, so they could be either modified or avoided.^25,57-59^ Also, typologies of PPPs have been suggested to try to improve accountability and transparency of their goverenace.^60^

 This study also sheds light on the advantages and importance that filing FOIs has for methodological and health policy research and to investigate transparency and accountability.

 Our findings suggest that public officials are less inclined to recognise that interactions with the F&BI or their allies might influence their own institutions’ policies and practices. For years, activists have been concerned about the political influence of the F&BI in the country.^56,61^ Still, people executing programs, bureaucrats, and beneficiaries rarely consider engaging with the F&BI for public health issues, mainly aiming to deliver programs to children, as problematic. Nevertheless in the past three years, some efforts to reduce industry interference in food policy-making are emerging in Mexico due to the unprecedented exhorts by the Human Rights Commission to protect children or the intersectoral GISAMAC (Grupo Intersectorial de Salud, Alimentación, Medio Ambiente y Competitividad) initiative promoting new legislation on healthy eating.^62^

 Although there have been concerns about PPPs for many years among scholars, public servants, and activists,^33,63^ no clear guidelines exist on how to face the CoI and governance issues in these arrangements. The existing ones are either published in collaboration with representatives of the food industry or are general guidelines for any type of engagement with the F&BI and with the tobacco industry.^40,64-66^ The use of core principles and practices of good governance is a way to move forward to identify the benefits and risks of PPPs. Likewise, when information is available, the use of the logical framework to identify information and key factors of any public program that should be available to the population (either on the institutional websites or if requested) might be helpful to improve the accountability and transparency of PPPs.

 PPPs are permanent in many settings but take more complex forms with F&BI participation through the third parties that they fund.^60^ However, the links with the industry are not made clear despite, in many cases, having a transparency platform that citizens can use, particularly in low-resource countries, making it more difficult for policy-makers and participants to perceive them as originating or being developed in collaboration with commercial actors. PPPs are endemic, and, in some instances, they might still be a good way of financing public health initiatives. For the type of PPPs studied here, the involvement of alternative industries other than the F&BI could serve as a solution to avoid institutional CoIs, promotion of harmful products, or breach of key principles of health governance in PPPs.^25,59^ Nevertheless, this will not be sufficient to change the problems that these PPPs have on transparency and governance unless they are accountable to third parties from their conception to their execution and results. As our results show, the lack of clear results on effectiveness was highly criticised and resulted in a lack of trust in public institutions. Our results show that governments aiming to advance the health agenda might benefit from preserving their independence, integrity and, therefore, credibility. As shown in this study, the private sector uses the public sector as a vehicle to reach the population they want and to set their own agenda, promoting their brands in attempts to keep loyal consumers.

 Even with the current strong mandates of the new Mexican government to avoid having formal relationships with the F&BI (at least at the Federal level under the SSA), there is still a need to further understand PPPs moving forward, given the power the F&BI has in the political and the economic situation of the country. It is essential that the academic community and public health advocates be vigilant and claim transparency of such partnerships, and advocate for them to end where necessary.

###  Limitations

 Whilst this study was designed and implemented using best practices available to us to address the study’s aims, we acknowledge the following limitations. First, by looking at individual PPPs in isolation, we may have neglected the cumulative and synergistic effect of PPPs, regardless of some of these being ethically problematic. Second, we collected data prior to and in the middle of the COVID-19 pandemic when global public health issues and challenges were occurring, thus limiting the availability of informants and responses from the government officials. This might have affected some views and answers of interviewees. Third, we had difficulties in tracking the information on PPPs, as there was no consistency in naming, reporting, monitoring, or talking about said PPPs. For the sake of this study, we used the title as we first encountered it in the literature and triangulated data, although it might have changed through time or by the time this paper is published. Finally, we acknowledge that the authors’ interest, nationality, and our expertise on the corporate political activity in Mexico’s food policy might have influenced the interpretation and assumptions on this exercise. Nevertheless, transparency on data collection and the analytical framework outweigh such limitations and supports the reliability and replicability of this research.

## Conclusion

 At both national and sub-national levels, the four PPPs studied in Mexico were found to have minimal public information available on their implementation, impact, or effectiveness in contributing to the public health agenda. The F&BI partner tended to dictate the design, information management, and implementation while promoting their brands through the partnerships. Few independent evaluations of the PPPs exist, and none reported on the effectiveness or relevance to public health. Good governance principles, such as accountability, transparency, fairness, participation, integrity, and credibility, were barely followed in each of the cases studied. CoI in such arrangements has not always been questioned by public officials, and if so, the risks of such PPPs did not always outweigh their financial benefits. This study shows that these PPPs produced minimal gains for public health while boosting the credibility of the participating transnational F&BI partners.

## Acknowledgements

 We would like to thank Jonathan Marks, Claudio Shuftan, and Margaret Miller for their valuable comments and suggestions to the project which this publication is based on.

## Ethical issues

 Ethics approval was obtained from the Comité de Ética en Investigación Instituto Nacional de Salud Pública on March 2, 2020.

## Competing interests

 Authors declare that they have no competing interests.

## Supplementary files


Supplementary file 1 contains (*a*) interview guide for experts and decision-makers and (*b*) coding framwork.
