# Supplementary material for: Public-Private Partnerships in Mexico: Implications of Engaging With the Food and Beverage Industry for Public Health Nutrition
Source: Int J Health Policy Manag. 2024 Feb 27;13:8008. doi: 10.34172/ijhpm.2024.8008 (PMC11016274; doi:10.34172/ijhpm.2024.8008)
Supplement: Supplementary file 1 — contains (a) interview guide for experts and decision-makers and (b) coding framwork. [file ijhpm-13-8008-s001.pdf]

Article title: Public-Private Partnerships in Mexico: Implications of Engaging With the Food and Beverage Industry for Public Health Nutrition

Journal name: International Journal of Health Policy and Management (IJHPM)

Authors' information: Angela Carriedo[1]*, Enaí Ojeda[2], Eric Crosbie[3,4], Mélissa Mialon[5]

[1]World Public Health Nutrition Association, London, UK.

[2]Instituto Nacional de Salud Pública, Morelos, Mexico.

[3]School of Public Health, University of Nevada Reno, Reno, NV, USA.

[4]Ozmen Institute for Global Studies, University of Nevada Reno, Reno, NV, USA.

[5]Trinity College Dublin, Dublin, Ireland.

***Correspondence to:** Angela Carriedo; Email: angela_carriedo@yahoo.com

Citation: Carriedo A, Ojeda E, Crosbie E, Mialon M. Public-private partnerships in Mexico: implications of engaging with the food and beverage industry for public health nutrition. Int J Health Policy Manag. 2024;13:8008. doi:10.34172/ijhpm.2024.8008

Supplementary file 1 contains (*a*) interview guide for experts and decision-makers and (*b*) coding framwork.

A. INTRODUCTION

I. Welcome and introduction.
II. Introduce the interviewer and clarify that notes will be taken during the interview.
III. Explain what the study consists of.
IV. Any feedback you give us is valuable. There are not right or wrong answers. They are all interesting experiences and opinions and that is what we want to know.
V. Read the consent.
VI. saw. Guarantee confidentiality of the information and that your testimony and personal data will be handled with great care.
VII. Indicate that the conversation will last approximately 60 minutes.
VIII. Request permission to be audio-recorded. (Start recording.)
IX. Confirm acceptance to participate and reiterate that the information from this interview is confidential.

B. ORIGIN AND PURPOSE OF THE PROGRAM/PROJECT

1. Do you know the origin of this program/project [Program Name]? Do you know which organizations/institutions or companies created it? Can you tell me a little more about this, please. (if you are familiar with several ask each for all questions)
2. Do you know when and how your organization/institution became involved in this [Program Name] programme/project?

3. Do you know what other organizations/institutions or companies are involved in this [Program Name] programme/project? Do you know if there are several collaborators (individuals, organizations/institutions, companies, government)?
4. How have you been involved in this programme/project?
5. Do you know what the objectives of this program/project [Program Name] are? What was it created for?
6. Are you aware of the services provided through this program/project?
7. In which states/localities/sites is this program/project being implemented?
8. Do you know who the program/project serves? (probe about the profile of people, eg students, teachers, rural communities, etc.). Is it known how many communities or people are served in total?

C. OPERATION AND FUNCTIONING OF THE PROGRAM/PROJECT

9. Do you know how different organizations/institutions are involved in the programme/project?
10. Can you tell me a little more about the operation and implementation of this project.
11. Do you know how long this project/program has been implemented?
12. Do you know what services or products the program/project provides? (For example, information, education, materials, products)
13. If this program/project provides any type of education or training, ask:

14.Do you know what topics are taught? (probe: information on nutrition, diet, obesity or diabetes, water: sanitation and water care, encourage water consumption, etc.) Could you tell us more about it? (if possible, probe: who designed the content, who delivers it, what is the model)

15. Do you think that the project/program has provided what was initially proposed?

D. EVALUATION AND MONITORING

16. Do you know who makes the decisions on how to operate this project/program? (Inquire about the collaborators mentioned or others)
17. Do you know how the responsibilities are divided between the collaborating organizations/institutions?
18. Do you know who evaluates or monitors that the activities/services reach the target population (the beneficiaries)? Can you tell me a little more about this.
19. Do you know if there are measures of accountability and transparency of the program/project? (e.g. adequate use of human, economic and material resources, etc.)
20. Do you know if the results of this program/project are public? For example, they are shared with the beneficiary population or published somewhere. If yes, where and who publishes them?

E. BRAND/COMPANY ASSOCIATIONS

21. Do you know if the people who receive the services or benefits of this program/project associate certain specific foods and drinks with this program/project?
22. Do you know if there are any brands associated with this project/program? Can you give me more details? (Inquire if the brand is: promoted, advertised, mentioned by implementers, etc.)?
23. The interviewer confirms the name of the company(s) involved in the project and asks the interviewer: What is her opinion that this brand(s) is(are) involved in this project?

F. BENEFITS AND WEAKNESSES OF THE PROGRAM/PROJECT

24. What are the benefits you perceive from this program? (Probe: education, health, other benefits to the community, investment/financial benefits, etc.)
25. What are the weaknesses that you perceive of this program/project?
26. Do you think that this program/project is helping to improve the health of the community? Because?
27. What are the benefits of carrying out programs/projects together with the government and the private sector?
28. What are the advantages for your organization/institution of this collaboration?
29. Do you think that this program/project could have a negative impact? Because?

G. PROSPECTS AND LIFE OF THE PROGRAMME/PROJECT

30. Do you know if this program will continue in the near future? Have you noticed changes since the new six-year term?
31. How do you think this program/project can be improved?
32. Do you think there are problems with this program/project (ethics, negative influence from the private sector)?
33. Could you provide me with more information about the program or tell me where I can find more information (documents or contacts)?

Supplementary material 1. Coding framework		
**Theme**	Codes	Descrption
**Structure (origin, goals, participan**	Origin of the program/project	Description about how the program or project started and the main reasons to have it (economic, political opportunity, SRA)
	How does this started	Describes how the relationship between the pubic and private entity started or how did the program came about.
	Organizations involved	Describes which organizations participated and when
	Goals and objectives of the program	Describes the main aim and objectives of the program or PPP
	Target audience of the program	Describes who was the program designed for
**Coverage/implementation**	Main services provided/ main activities/ practices and how do they have worked throughout the program.	This code explains the perceptions or the description made about how the program operates and worked.
	Brand loyalty/association	Describes if the beneficiaries perceived any marketing or promotion by the private entity throughout the execution, promotion of the program, or if any of the partners´ aims or means considered this practice.
**Evaluation and monitoring**	Monitoring and indicators	Describes whether the interviewee or the document has any information about the program´s monitoring system or has executed it, and which indicators were considered.
	Evaluation reports	Describes the mechanisms of evaluation of the report, who was in charged of executing them and if they were/are publicly available.
	Executer	Describes who was in charged of the evaluation and monitoring of the program.
**Impact**	Perceived positive and negative impacts	Describes the interviewee´s opinion or the document´s content about the impacts (negative or positive) of the PPP/program
**Governance**	Decision makers involved/key public and private partners	Describes who was involved in making the decision about the program.
	Transparency mechanisms	Describes how the transparency mechanisms have been achieved (or not) throughout the partnership/program
	Decision making process and principles followed	Describes what were the main principles followed (if they were or are described in the docs).
	Governance mechanisms/structure	Describes the governance mechanisms, participants and responsibilities of each one
	Accountability mechanisms	Describes the accountability mechanisms for this particular program or agreement (or the lack of them).
	Coercive or voluntary (I am not sure what you mean here)	Describes whether the partnership or agreement has/had a principle to report results and processes of the program/partnership, or if it is done on a voluntary basis.
	COI guidelines	Describes if the PPP or the program follows COI guidelines or principles to protect public health goals.
**Continuity of the program**	Continuity of the program	Describes what has happened with the program/PPP to date (termination, continuity, changes, etc.) and factors associated to these changes.
	Improvements to be made	Describes the perceived benefits to be made (to the program OR to programs working like this one, or in general PPPs)
**Externalities of the PPP**	Externalities	Describes what are the main implications perceived of this PPP (or in general, PPPs) to public health (aims, programs, governance, etc.)
**Threats**	to the person	
	to the institution	
	to the program	Describes the perceived threats to the person (interviewee), the institution or the program.
**Weaknesses and benefits**	Perceived benefits of the program	Describes the interviewee´s opinion or the document´s content about the benefits of the program.
	Perceived weaknesses of the program	Describes the interviewee´s opinion or the document´s content about the weaknesses of program.
	Perceived benefits/strengths o thef PPP	Describes the interviewee´s opinion or the document´s content about the benefits of the PPP described.
	Perceived weaknesses of the PPP	Describes the interviewee´s opinion or the document´s content about the weaknesses of the PPP described.
	Perceived benefits for the institution /association	Describes the interviewee´s opinion or the document´s description about the benefits the PPP had for their organization/association
	Perceived weaknesses for the institution/association	Describes the interviewee´s opinion or the document´s content about the weaknesses PPP had for their organization/association
